# Supplementary material for: Correlation between Adrenoceptor Expression and Clinical Parameters in Degenerated Lumbar Intervertebral Discs
Source: Int J Mol Sci. 2022 Dec 5;23(23):15358. doi: 10.3390/ijms232315358 (PMC9739018; doi:10.3390/ijms232315358)
Supplement: Supplementary file 1 [file ijms-23-15358-s001.zip › ijms-1922086-Supplementary.pdf]

Supplementary material:

Table S1. Medians of expression values with interquartile ranges for the respective ARs and groups

Receptor	Group	Median	25 percentile	75 percentile
α1A	Modic 0-I	0,29	0,14	0,67
	Modic II-III	0,76	0,30	1,14
α2A	Modic 0-I	0,08	0,04	0,42
	Modic II-III	0,40	0,18	0,63
β2	Modic 0-I	0,29	0,15	0,95
	Modic II-III	0,76	0,41	1,03
α1A	Pfirrmann II	0,07	0,00	0,14
	Pfirrmann III	0,29	0,16	0,67
	Pfirrmann IV	0,73	0,32	0,99
	Pfirrmann V	0,75	0,30	1,44
α2A	Pfirrmann II	0,04	0,00	0,08
	Pfirrmann III	0,17	0,04	0,42
	Pfirrmann IV	0,33	0,17	0,90
	Pfirrmann V	0,50	0,18	0,81
β2	Pfirrmann II	0,17	0,07	0,26
	Pfirrmann III	0,29	0,15	0,63
	Pfirrmann IV	0,73	0,62	1,03
	Pfirrmann V	0,76	0,35	1,44
α1A	NPRS < 7.5	0,74	0,41	1,12
	NPRS ≥ 7.5	0,23	0,13	0,77
α2A	NPRS < 7.5	0,36	0,12	0,62
	NPRS ≥ 7.5	0,19	0,06	0,61
β2	NPRS < 7.5	0,78	0,31	1,31
	NPRS ≥ 7.5	0,42	0,21	0,77
α1A	◎-Blocker	0,30	0,16	0,62
	No ◎-Blocker	0,76	0,34	1,50
α2A	◎-Blocker	0,20	0,08	0,41
	No b-Blocker	0,53	0,09	0,79
β2	◎-Blocker	0,36	0,15	0,76
	No ◎-Blocker	0,81	0,33	1,19
α1A	Nicotine	0,63	0,21	1,51

	No Nicotine	0,45	0,19	0,90
α2A	Nicotine	0,17	0,00	0,81
	No Nicotine	0,30	0,09	0,57
β2	Nicotine	0,69	0,30	1,28
	No Nicotine	0,58	0,18	0,93
α1A	NSAID	0,73	0,28	1,11
	No NSAID	0,44	0,16	0,75
α2A	NSAID	0,45	0,17	0,75
	No NSAID	0,17	0,07	0,42
β2	NSAID	0,77	0,55	1,08
	No NSAID	0,31	0,19	0,81
α1A	ASD	0,18	0,14	0,28
	No ASD	0,62	0,25	0,98
α2A	ASD	0,42	0,08	1,00
	No ASD	0,22	0,07	0,57
β2	ASD	0,50	0,07	0,86
	No ASD	0,63	0,26	0,99

NPRS - Numeric pain rating scale; NSAID - Non-steroidal anti-inflammatory drugs ; ASD - Adjacent segment disease

Figure S1:

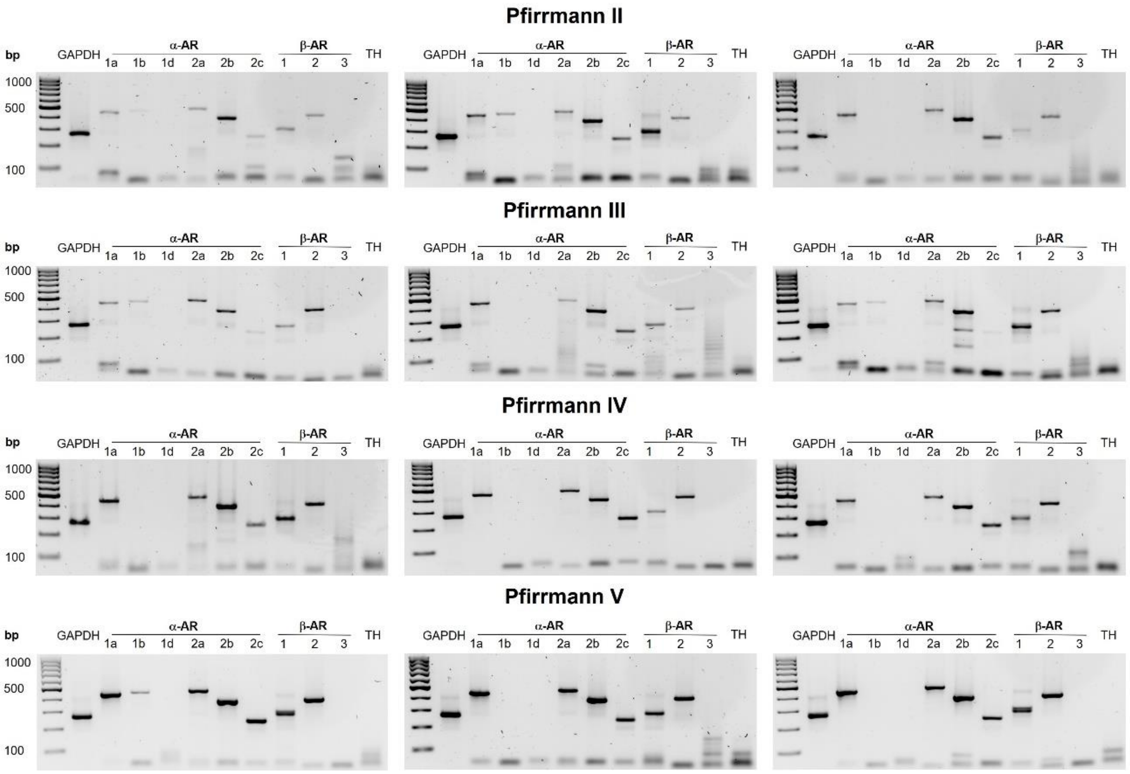

Figure S1. Adrenoceptor (AR) and tyrosine hydroxylase (TH) gene expression in human intervertebral disc tissue determined by RT-PCR. GAPDH was used as a housekeeping gene for normalization. RT-PCR gels were analyzed densitometrically based on the intensities of corresponding PCR bands. GAPDH band intensity of each individual patient was defined as "1" and AR expression levels were calculated in relation to that value.

Figure S2:

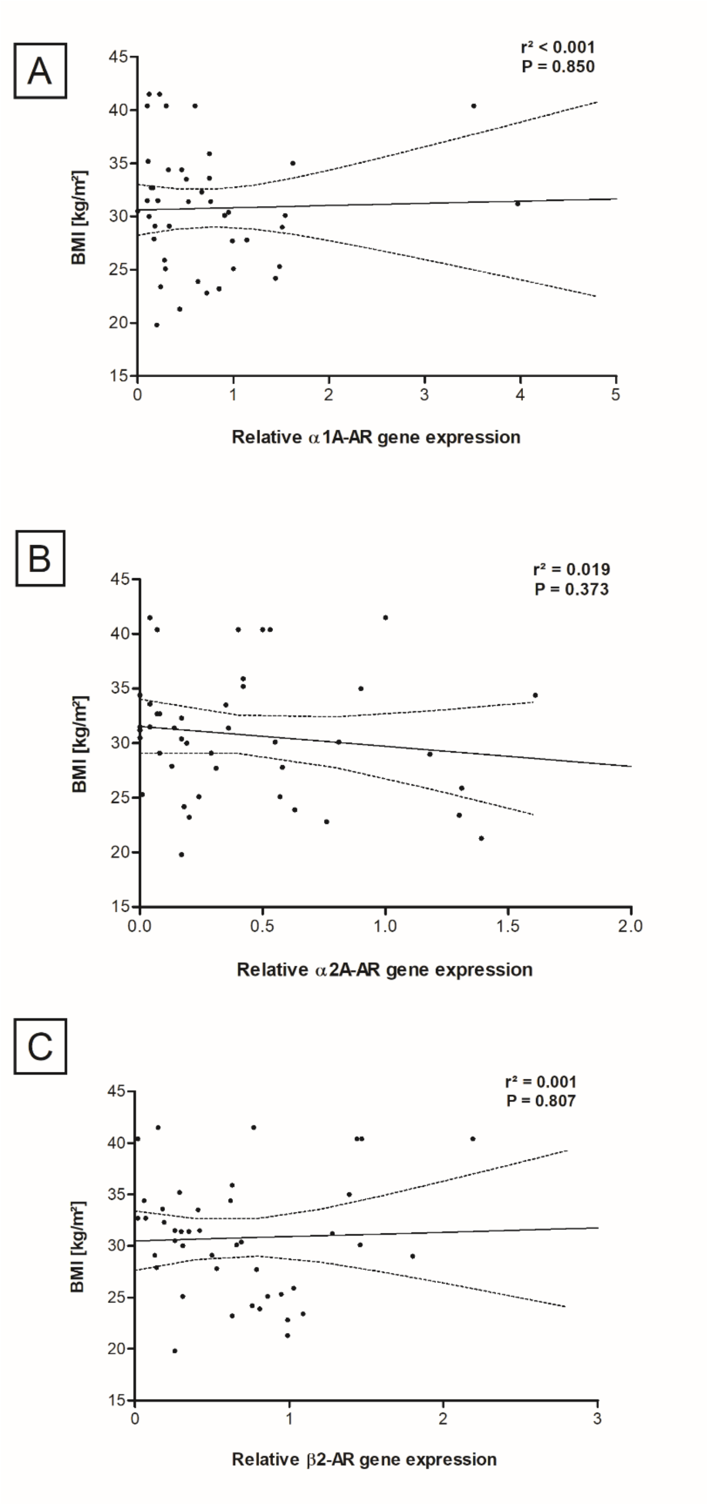

Figure S2. BMI and relative AR gene expression. Scatter diagrams of the relationship between BMI and relative a1A-AR (A), a2A-AR (B) and B2-AR gene expression (C). There was no correlation between BMI and AR expression. Each black dot represents an individual patient. Data represent medians with interquartile range.

Figure S3:

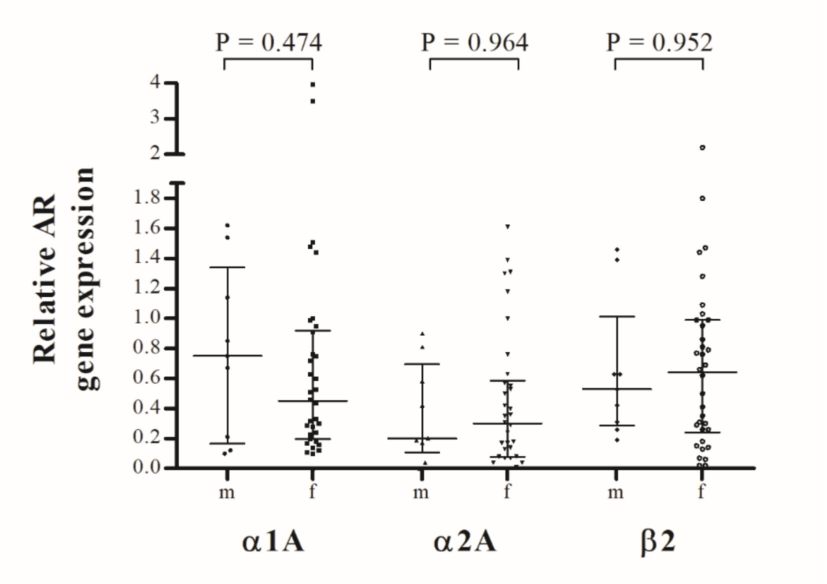

Figure S3. Interrelationship between relative α1A-, α2A-, and β2-AR gene expression and sex (m – male; f – female). Each black dot represents an individual patient (n=43). Data represent medians with interquartile range.
